# Finely tunable dynamical coloration using bicontinuous micrometer-domains

**DOI:** 10.1038/s41467-022-31020-0

**Published:** 2022-06-24

**Authors:** Yuyin Xi, Fan Zhang, Yuanchi Ma, Vivek M. Prabhu, Yun Liu

**Affiliations:** 1 Center for Neutron Research, National Institute of Standards and Technology, Gaithersburg, MD 20899 USA; 2 Department of Chemical & Biomolecular Engineering, University of Delaware, Newark, DE 19716 USA; 3 Materials Measurement Science Division, National Institute of Standards and Technology, Gaithersburg, MD 20899 USA; 4 Materials Science and Engineering Division, National Institute of Standards and Technology, Gaithersburg, MD 20899 USA; 5 Department of Physics & Astronomy, University of Delaware, Newark, DE 19716 USA

**Keywords:** Gels and hydrogels, Self-assembly, Metamaterials

## Abstract

Nanostructures similar to those found in the vividly blue wings of Morpho butterflies and colorful photonic crystals enable structural color through constructive interference of light waves. Different from commonly studied structure-colored materials using periodic structures to manipulate optical properties, we report a previously unrecognized approach to precisely control the structural color and light transmission via a novel photonic colloidal gel without long-range order. Nanoparticles in this gel form micrometer-sized bicontinuous domains driven by the microphase separation of binary solvents. This approach enables dynamic coloration with a precise wavelength selectivity over a broad range of wavelengths extended well beyond the visible light that is not achievable with traditional methods. The dynamic wavelength selectivity is thermally tunable, reversible, and the material fabrication is easily scalable.

## Abstract (web summary)

Structural colors are often produced by periodic structured materials leading to the constructive interference of light waves. Here, the authors report control of structural color and light transmission via a colloidal gel and dynamic coloration with a precise wavelength selectivity over a broad range of wavelengths by taking advantage of the Christiansen effect.

## Introduction

Ever since ancient Mesopotamia, color has occupied a unique place in human civilization. In the past decades, photonic devices have attracted substantial attention due to their ability to control light flow, with applications such as structural coloration^1^, smart windows^2^, optical switches^3^, reflective displays^4^, and memories^5^.

Among various optical materials, structure-colored materials have generated broad interest due to their versatility of color control, vivid color effects, long-term durability, and resistance to photobleaching^6,7^. They are also commonly found in natural materials, such as bird feathers and flower petals^8,9^. Structural color results from the pure physical interaction between light and materials (in contrast to absorption), where light does not exchange energy with a sample^10^. Light scattering, reflection, and deflection are typical processes that are commonly observed in structural color^10^. In many structure-colored materials, solid building blocks (e.g., particles) need to be arranged into periodical structures with dimensions comparable to the wavelength of visible light (hundreds of nanometers). Thus, the dynamic coloration of these structure-colored materials is conventionally achieved by altering the structure size to control the wavelength of scattered light. The responses to stretching^11,12^, temperature^13^, moisture/UV^14^, and PH^15^ can directly translate to color change. However, the extent of these responses is often limited, placing a stringent constraint on the materials’ sensitivity and tunable wavelength range. Structural color via controlling transmitted lights represents a significantly less explored alternative to scattering^16–18^, despite its advantage in many vital applications, such as smart windows, optical filters, and displays^7,19–22^. Some recent applications of structure-colored materials based on the Christiansen effect to realize dynamic tunability in transmitted light are also limited in terms of the range and flexibility of the tunability^17,18,23–26^.

We report here a previously unrecognized approach that precisely controls the structural color and light transmission via a novel photonic colloidal gel without long-range order. It also enables finely adjustable dynamic color response over an extensive range of wavelengths extending well beyond the visible light spectrum. It is a general method to manage light transmission with a narrow transmitted bandwidth. In addition, the material fabrication is based on the kinetically trapped phase separation of the binary solvent by nanoparticles. Thus, this approach is versatile with great tunability and the process can be easily scaled up. It is a promising candidate for use in various stimuli-responsive photonic devices and may inspire new classes of structural-color materials.

## Results and discussion

### Dynamically tunable coloration of SeedGel

Nanoparticles dispersed in a binary solvent can thermo-reversibly assemble into solid bicontinuous structures by forming solvent segregation-driven gel (SeedGel) in a scalable, reproducible, and tunable manner^27,28^. Different from bicontinuous interfacially jammed emulsion gels (Bijels), where particles have neutral wettability for both components in a binary solvent, particles in SeedGel favor one component of a binary solvent that results in gels consisting of alternating micrometer-sized bicontinuous domains: the particle domain and the solvent domain.^27,29^. Particles are jammed in the particle domain, which facilitates the enrichment of the solvent component that particles favor. (A schematic picture of a SeedGel is shown in Supplementary Fig. 1.)

**Fig. 1:**
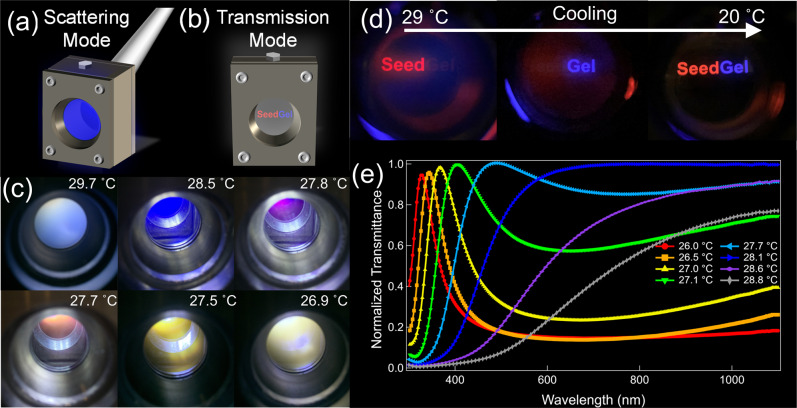
Dynamically tunable coloration of SeedGel in both scattering mode and transmission mode. The schematic drawing illustrates the experimental setup in **a** scattering mode and **b** transmission mode. Photographs of SeedGel with **c** tunable structural color recorded in scattering mode at different temperatures and **d** temperature-dependent light transmission with different colors in transmission mode. A white light source is used in (**c**) and the colored word ‘SeedGel’ in red and blue in (**d**) is displayed on an OLED (organic light-emitting diode) screen. **e** Normalized transmittance of the SeedGel as a function of wavelength at different temperatures. An increase in temperature shifts the transmission peak towards a longer wavelength.

Here, we show that the dynamically tunable coloration can be realized based on unique properties of the SeedGel framework with micrometer-sized domains, whose underlining mechanism can be a general approach for many other optical materials. We first demonstrate this with a model SeedGel system of charged silica nanoparticles (27 nm in diameter) dispersed in a binary solvent of 2,6-lutidine and water. This dispersion becomes a solid gel (SeedGel) above 26 °C, with tortuous micrometer-sized bicontinuous domains^27^. Below 26 °C, the dispersion is a transparent liquid. To demonstrate the dynamically tunable color, we designed an experimental setup with both a scattering mode and a transmission mode, as illustrated in Fig. 1a, b. In the scattering mode, we placed a white-light source on one side of a sample and positioned the camera off at an angle on the other side to capture the scattered light. Figure 1c shows that the sample remains transparent while its color transitions from blue to yellow when the temperature decreases from 28.5 to 27.5 °C but becomes increasingly opaque for temperatures above 29.7 °C or below 26.9 °C.

In the transmission mode, we placed red and blue light on one side of the sample and a camera on the opposite side to capture the transmitted light. The camera is primarily sensitive to the transmitted light that is much more intense than the scattered light. The wavelength of the transmitted light is complementary to that of the scattered light (Fig. 1d). Long-wavelength (red) light passes through the SeedGel at high temperatures, and short-wavelength (blue) light is permitted at low temperatures. (Supplementary Movie 1 recorded the cooling process) At temperatures below the gelation point (26 °C), the SeedGel reverts to a liquid sample (20 °C), transparent to all visible light. (Details are shown in Supplementary Fig. 2.) The coloration is not a result of absorption or luminescence of the chemical species, but due to the structures formed within the sample.

To quantify the temperature-dependent optical properties of this material, we recorded the light transmittance as a function of wavelength using a UV–Vis spectrometer. The transmittances at different temperatures in Fig. 1e are normalized by that measured at 20 °C.

At 26 °C, the light transmission shows a peak at 327 nm (ultraviolet) with almost 100% normalized transmittance at the peak position and a full width at half maximum (FWHM) around 67 nm. Thus, the SeedGel is transparent only for the light within this narrow band and opaque for light at other wavelengths. Because the peak wavelength is outside the visible light spectrum, the material appears opaque to human eyes, consistent with the results in Fig. 1c that the sample appears turbid below 26.9 °C. Increasing temperature shifts the transmittance peak position to a longer wavelength, making the material transparent to visible light, as captured in Fig. 1c, d. The peak shift can be finely adjusted by precise control of the sample temperature. Eventually, at ≈29 °C, the sample is turbid again because the transmission peak shifts to the infrared regime.

The well-defined peaks at temperatures between 26 and 28 °C offer a high-precision wavelength selection by tuning temperature. The FWHM of the transmittance peak becomes larger at higher temperatures (in other words, the peak becomes broadened at higher temperatures) but is controllable as demonstrated and explained later in this paper. The transmittance is tunable not only for the entire visible light spectrum but also for the ultraviolet and infrared regions. Supplementary Fig. 3 shows that the modulation of near-infrared light reaches up to about 2500 nm in wavelength. Such control can be pivotal in sensing and managing heat transfers. The extensive wavelength tunability of the SeedGel is truly unique among all structural coloration materials and opens up possibilities to applications that require good control of the transmitted light.

### Structure characterizations

**Fig. 2:**
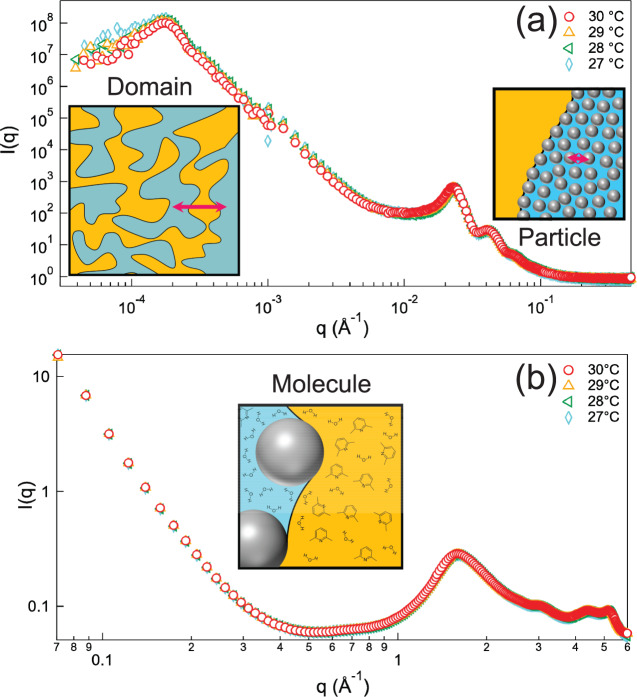
Structure characterizations in multi-length scales using scattering techniques. The scattering profiles of SeedGel within the temperature range exhibiting tunable structural color characterized by **a** USANS/SANS and **b** SAXS/WAXS. The inset images show the schematic drawing of the structures at different length scales probed by different *q*-ranges. The solvent compositions in the particle-rich and solvent domains are different from each other and they are denoted with different colors. The error bars represent one standard deviation and are often smaller than the symbol size.

In many conventional structurally colored materials based on reflected and scattered lights, the scattering/reflection of visible light is typically related to structures with repeating distances comparable to the light wavelength^30^. However, in the SeedGel, such sub-micrometer periodical structures are absent, as shown by the neutron and X-ray scattering data over a wide *q*-range (Fig. 2) that probes the structure from sub-angstrom to ≈20 µm. Here, *q* is the magnitude of the scattering wave vector.

The data of small-angle neutron scattering (SANS) and ultra-SANS (USANS) are shown in Fig. 2a covering the temperature range that SeedGel displays visible color. The peak at *q* near 1.7 × 10^−4^ Å^−1^ indicates that the repeating distance of the bicontinuous domain in our sample is ≈3.4 µm, significantly greater than the wavelength of visible light (Supplementary Fig. 4). The peak at high-*q* (*q* ≈ 0.023 Å^−1^) corresponds to an average inter-particle distance of ≈30 nm due to the random packing of particles in the particle domain, much smaller than the wavelength of visible light. As the size distribution of the silica nanoparticles is fairly narrow, scattering peaks of the form factor of the sphere at higher *q*-positions are also visible at *q* ≈ 0.041 Å^−1^ and *q* ≈ 0.065 Å^−1^. It is also worth noting that the scattering patterns in Fig. 2a, b have no significant change within the temperature range where the sample exhibits dramatic color change. The fitting of the peaks in both the USANS region and SANS region suggests that the inter-domain distance stays at 3.4 μm and local particle concentration remains at ≈40% within the temperature range of interest (Supplementary Fig. 4 and Supplementary Table 1). Because the silica particles dominate the scattering intensity, this result indicates that the dynamical coloration is not due to a structural change of the silica particle domain. Small-angle X-ray scattering (SAXS) data (Supplementary Fig. 5) and wide-angle X-ray scattering (WAXS) data in Fig. 2b further confirm that neither the nanoparticles nor the solvent molecules form crystal structures. The combination of X-ray and neutron scattering data decisively demonstrates that the SeedGel has a different mechanism of light control based on periodic structures.

### Mechanism of dynamically tunable coloration

While the particle domain structure of the SeedGel remains stable within the studied temperature range, the solvent compositions in bicontinuous domains depend on the temperature due to thermodynamics. Above the gel temperature, the binary solvent in a SeedGel separates into two phases: a lutidine-rich phase and a water-rich phase. In the gel state, the lutidine-rich phase forms the solvent domain^27^. The water-rich solvent phase fills the spaces inside the particle domain because a highly charged particle surface favors water. A change in temperature affects the solvent composition in both domains. An increase in temperature reduces the lutidine concentration in the particle domain and consequently increases the lutidine concentration in the solvent domain. The mass fraction of lutidine in the particle domain decreases from ≈15% at 27.5 °C to <10% at 30 °C in our sample. (See details in Supplementary Fig. 6 and Supplementary Note 5.) The dynamic change of solvent compositions in both domains would affect their averaged scattering length density, which leads to slight intensity changes at different temperatures in the USANS region (Fig. 2a).

The solvent exchange between two domains offers a sensitive control of the light transmission through the material. Light scattering in SeedGel affects the light transmittance and is controlled by the difference of the refractive index between the particle and solvent domains. The refractive index of lutidine (≈1.49) is comparable to that of silica (≈1.47) but much higher than that of water (≈1.33). Because the solvent compositions of these domains depend on the temperature, the component-averaged refractive indices of both domains are thermo-reversibly tunable even though the overall domain structures remain stable.

**Fig. 3:**
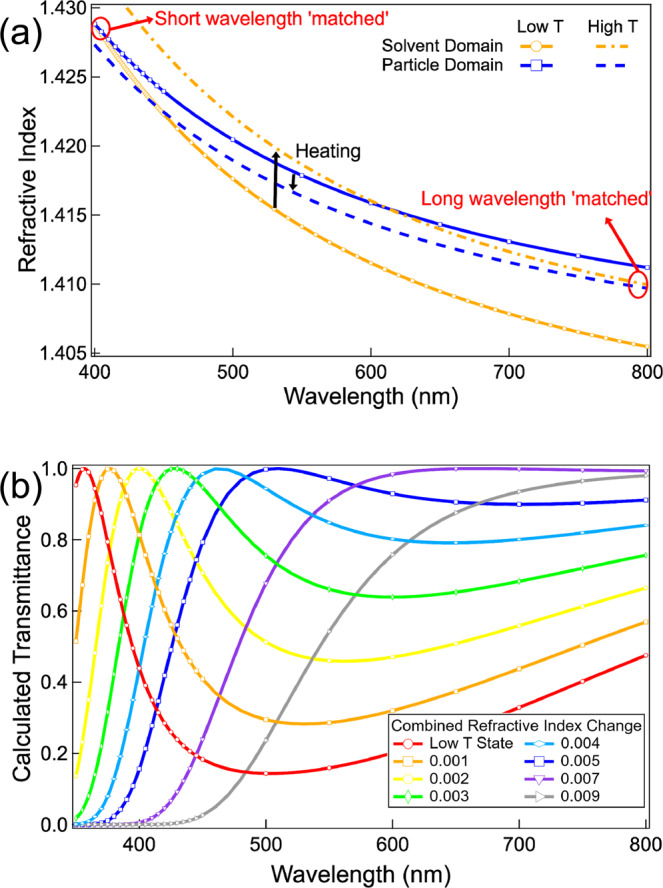
Wavelength-dependent refractive index and transmittance. **a** Calculated refractive indices of both particle and solvent domains in response to the temperature change. The low-T state refers to the point with a similar refractive index between the two domains at a wavelength around 400 nm. A temperature rise (high-T state) increases the refractive index of the solvent domain and decreases that of the particle domain, making the refractive index at a long wavelength (around 800 nm) ‘matched’. **b** Calculated transmittance of SeedGel as a function of wavelength at different temperatures. A combined refractive index change of 0.007 between the two domains is enough to shift the ‘matched’ wavelength across the full visible range. The refractive index of both the particle and solvent domains that lead to each calculated transmission is shown in Supplementary Fig. 10.

It turns out that the wavelength selection of the SeedGel is due to the different wavelength-dependence of the refractive indices of the solvent and particle domains, which is also called the Christiansen effect^31^. Lights with the selected wavelength can pass through a sample only when the refractive indexes of two domains in a strong scattering sample match at a chosen wavelength. To demonstrate this, we estimated the wavelength-dependent refractive index of the particle and solvent domains. (See details in Supplementary Figs 7 and 8). Figure 3a shows the calculated domain-specific refractive indices in the wavelength from 400 to 800 nm. At a low temperature, the refractive indices of the particle domain (solid blue line) and solvent domain (solid orange line) match at a short wavelength (≈400 nm). The sample is thus transparent to blue light at low temperatures, as shown in Fig. 1d. At the same time, the refractive index difference becomes larger at a longer wavelength. As the SeedGel has micrometer-sized bicontinuous domains, a very small deviation of the refractive indices between these two domains results in strong scattering and low light transmission.

Upon heating, the lutidine concentration rises in the solvent domain, leading to a refractive index increase. In contrast, the decrease of the lutidine concentration in the particle domain lowers its averaged refractive index. As a result, at a high temperature, the refractive index of the particle domain (dashed blue line) matches that of the solvent domain (dashed orange line) at a longer wavelength. The temperature-induced refractive index change of both domains is also evidenced by the change of contrast (scattering intensity) in static light scattering experiments (Supplementary Fig. 9). The unique combination of micrometer-sized domains and fine control of the solvent composition using the binary solvent fully elucidates the fascinating optical properties of the SeedGel. We further estimate the transmittance semi-quantitatively (Supplementary Fig. 10). Figure 3b shows the calculated transmittance by varying the relative refractive index between the two domains with excellent agreement with the experimental trend shown in Fig. 1e. The refractive indexes of both domains change sharply at the short-wavelength region than that at the long-wavelength region. It explains the sharp peak transmittance at the short wavelength region and broad peak transmittance at the long-wavelength region. Interestingly, as the light transmittance of a material is proportional to ${e}^{-{(\triangle \rho )}^{2}{Ad}}$, where *d* is the material thickness, $\triangle \rho$ is the refractive-index difference between two domains, and *A* is a constant related to the domain structure, increasing *d* can make the transmission peak much narrower and sharper. The experimental results in Supplementary Fig. 11 show that increasing the path length reduces the FWHM to 44 nm which is comparable to that of quantum dots^7^ and agrees with the calculated result (Supplementary Fig. 12).

### A versatile platform for optical applications

The binary solvent exchange in SeedGels provides a previously unrecognized way to manipulate materials for the desired color properties. Previous materials using the Christiansen filters often directly rely on the refractive index change of the sample (macromolecules, solvents, or liquid crystals) in response to external stimuli (e.g. temperature)^17,18,23–26,32^. Here, the adjustable concentration of solvent molecules in the two phases is responsible for the dynamically tunable transmission in SeedGel. This fundamental difference renders several unique characteristics in SeedGel. First, it becomes possible to customize the transition temperature in SeedGel, which is otherwise not trivial to achieve. Second, the wavelength modulation of SeedGel spans a wide range from ultraviolet to infrared range, much broader than that of materials previously reported^17,18,23–26,32^. Third, the absolute peak transmittance realized through the SeedGel approach is fairly high (about 90%, Supplementary Fig. 2). This is due to the highly uniform structures formed in each domain, and the large transmittance of individual components in a SeedGel. In addition, as a SeedGel is thermo-reversibly tunable, a gel can be reversed back to a liquid system transparent to all colors as needed, providing additional optical control for a SeedGel sample.

**Fig. 4:**
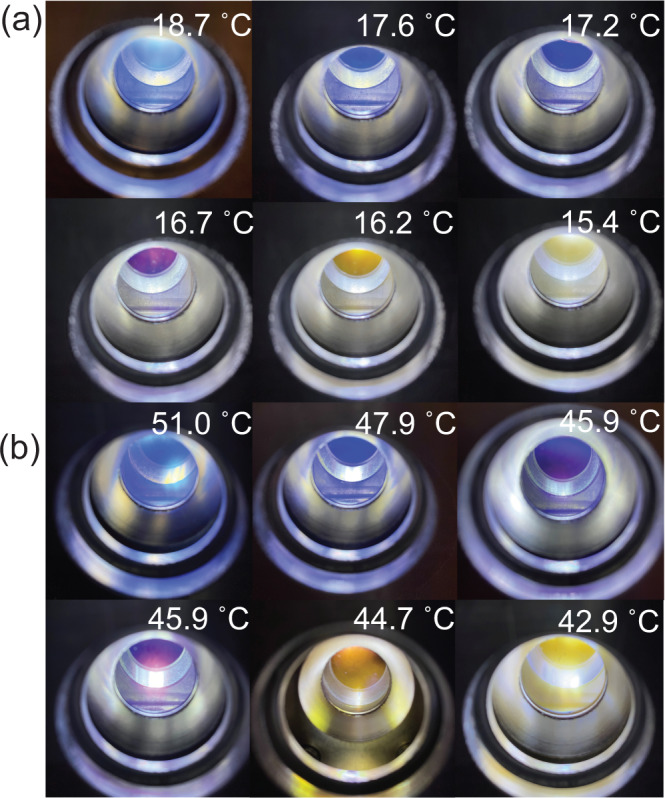
The customizable color transition temperature realized by different binary solvents. SeedGel prepared with binary solvents of **a** water/2,4-lutidine and **b** water/3-methylpyridine shifts the color transition point to lower and higher temperatures, respectively, when compared to that of SeedGel prepared with water/2,6-lutidine.

A few examples are demonstrated here to show the flexibility offered by the SeedGel approach to customize the material to meet the needs of different applications. By using different binary solvents, we can adjust the gelation temperature, thus the operating temperature range for the observed optical properties. Figure 4 demonstrates SeedGels formed with two different types of binary solvents: water/2,4-lutidine and water/3-methylpyridine. For water/2,4-lutidine (an isomer of 2,6-lutidine), the color transition temperature can be lowered to below 20 °C, while for water/3-methylpyridine, the color transition temperature starts at ≈50 °C. The transition temperature can be further increased to ≈60 and ≈80 °C by using water/deuterated 3-methylpyridine and water/2-methylpyridine, respectively. The corresponding transmission measurements with these solvent pairs are shown in Supplementary Fig. 13. As SeedGels can be prepared by different types of particles and different binary solvents, SeedGels thus offer great flexibility to form materials with desired optical properties.

In conclusion, we discovered a previously unrecognized approach to achieve tunable light scattering and transmission from micrometer-sized domains without long-range order. The SeedGel demonstrated here offers precisely tunable light transmission with a narrow wavelength bandwidth. And its transmission peak is adjustable in a very wide range of a wavelength spectrum. Using the binary solvent, a fully thermoreversible dynamic coloration over a broad spectrum is realized. It is scalable because the kinetic arrest of colloidal particles allows for quasi-static ramping (0.1 °C/min). Its mechanism for tunable optical property opens a door to a new library of materials as photonic devices, including smart windows, optical fibers, and sensors.

## Methods

### Materials

Solvents and chemicals were used as received without further purification. 2,4-Lutidine (99%), 2,6-Lutidine (99%), 3-methylpyridine, and silica nanoparticle dispersion (Ludox TM, mass fraction of 50%) were purchased from Sigma-Aldrich (St Louis, MO, USA).

### Preparation of SeedGel

To prepare the material shown in Fig. 1, we mixed every milliliter of highly-charged silica-nanoparticle dispersion in water (Ludox TM) with 300 μL 2,6-lutidine at room temperature. A physical gel was formed immediately upon mixing. Vigorous vortexing or continuous rolling was applied to the sample to ensure sufficient mixing. Consequently, a homogenous solution was obtained at low temperatures. Upon heating, a sample became a solid gel (SeedGel) that showed the structural color and tunable optical transmission at elevated temperatures. By cooling the sample to 4 °C, the sample became a homogeneous transparent liquid. To vary the transition temperature of the SeedGel (materials shown in Fig. 4) to control the functional temperature range of optical effect, we replaced 2,6-lutidine with 2,4-lutidine or 3-methylpyridine. To prepare sample with 2,4-lutidine, every milliliter of highly-charged silica-nanoparticle dispersion in water (Ludox TM) was mixed with 300 μL 2,4-lutidine. The solution was vortexed and stored at 4 °C to ensure mixing into homogeneous solution. To prepare sample with 3-methylpyridine, every milliliter of highly charged silica-nanoparticle dispersion in water (Ludox TM) was mixed with 400 μL 3-methylpyridine. Vortexing and continuous rolling were applied to the sample at room temperature to facilitate well-mixing.

### UV–vis spectroscopy

A Thermo Scientific Evolution 201 UV–VIS Spectrometer (Waltham, MA, USA) was used to record the optical transmission spectrum as a function of temperature. A cuvette with a path length of 1 mm was used. The temperature was controlled by a Peltier element connected to a thermal bath. For each temperature change, a sample was equilibrated for at least 15 min before each measurement. A Cary 5000 (Agilent, Santa Clara, CA, USA) spectrometer was used to extend the measurement to the near-infrared range.

### SANS and USANS

Both SANS and USANS experiments were conducted at the NIST Center for Neutron Research (NCNR, Gaithersburg, MD, USA). 30 m SANS instrument (NGB-30) was used to obtain the SANS profiles^33^. Scattering patterns from three detector positions were stitched together to access a full *q*-range between 1 × 10^−3^ and 0.45 Å^−1^. Demountable titanium cells with quartz windows and a path length of 1 mm were used to hold the sample. The temperature of the sample environment was controlled by a Peltier module. USANS experiment was conducted on the USANS instrument at NCNR (BT-5), which covers a *q*-range of 3 × 10^−5 ^Å^−1^ < *q* < 1 × 10^−3^ Å^−1^. A circulation bath was hooked up to a multi-position sample changer (6CB) to regulate the temperature. Both the SANS and USANS results were reduced with Igor macros (Wavemetrics, Portland, OR, USA)^34^.

### SAXS and WAXS

We performed in situ USAXS, SAXS, and WAXS measurements at the USAXS beamline at Advanced Photon Source, Argonne National Laboratory (Lemont, IL, USA)^35^. The combined, continuous *q*-range encompasses 1 × 10^−4^ to 6 Å^−1^. 21 keV X-ray was used for the measurements. The temperature was controlled by Linkam (Tadworth, UK) THMS600 controller with a temperature precision of 0.1 °C. The data were reduced using Igor Pro-based Indra, Irena, and Nika packages^36^.

## Supplementary information

Supplementary Information

Description of Additional Supplementary Files

Supplementary Movie 1

## Data Availability

The data that support the findings of this study are available from the corresponding author.
