# Supplementary material for: Finely tunable dynamical coloration using bicontinuous micrometer-domains
Source: Nat Commun. 2022 Jun 24;13:3619. doi: 10.1038/s41467-022-31020-0 (PMC9232638; doi:10.1038/s41467-022-31020-0)
Supplement: Supplementary file 1 — Supplementary Information [file 41467_2022_31020_MOESM1_ESM.pdf]

Supplementary Information

Finely Tunable Dynamical Coloration Using Bicontinuous Micrometer-domains

Yuyin Xi [1,2], Fan Zhang [3], Yuanchi Ma [4], Vivek M. Prabhu[4] and Yun Liu [1,2,5*]

1. Center for Neutron Research, National Institute of Standards and Technology, Gaithersburg, MD, 20899, USA
2. Department of Chemical & Biomolecular Engineering, University of Delaware, Newark, DE, 19716, USA
3. Materials Measurement Science Division, National Institute of Standards and Technology, Gaithersburg, MD, 20899, USA
4. Materials Science and Engineering Division, National Institute of Standards and Technology, Gaithersburg, MD, 20899, USA
5. Department of Physics & Astronomy, University of Delaware, Newark, DE, 19716, USA

Correspondence to: yun.liu@nist.gov/ yunliu@udel.edu

Supplementary Notes:

1. **Schematic illustration of the structures in a SeedGel.**

A sample made of nanoparticles dispersed in a binary solvent can form a solvent segregation driven gel (SeedGel) upon moving the sample temperature close to the critical point of the binary solvent. A SeedGel consists of alternating particle and solvent domains as schematically shown in Supplementary Fig 1. [1,2] As marked in the figure, the light gray regions in Supplementary Fig 1 are the particle domain while the blue regions are the solvent domain. One model system discussed in this paper is made of silica nanoparticles in a mixture of water and 2,6-lutidine. By heating this sample, it can form a SeedGel. As shown in the zoomed-in image, jammed silica nanoparticles are surrounded by the water-rich solvent in the particle domain, and the solvent domain is made of the lutidine-rich solvent. In this model system, the diameter of particles is about 30 nm. And the size of the domains is a few micrometers. The highly charged nanoparticles are uniformly jammed within the particle domain (Supplementary Fig 1).

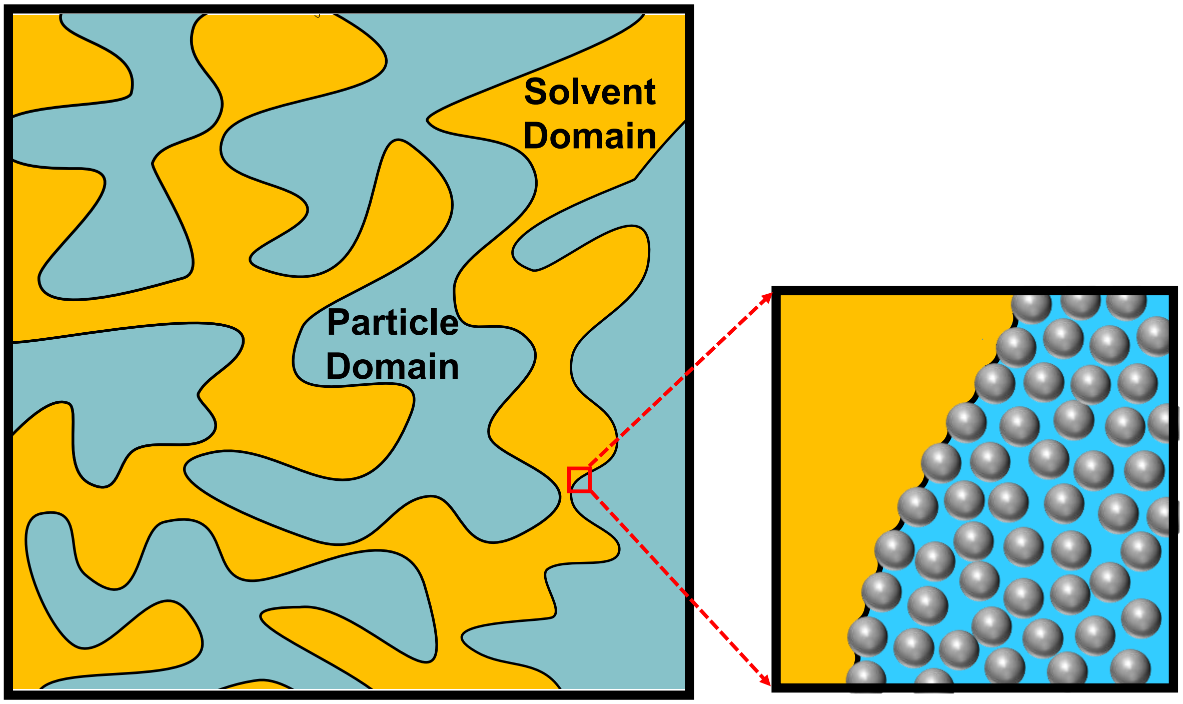

Supplementary Fig 1. The schematics of the bicontinuous domains formed by nanoparticles in SeedGel.

2. The sample is in a liquid state at 20˚C and is transparent to all visible light.

The chemical components of the sample shown in Figure 1 of the main text have neither strong light absorption (extinction) nor optical emission within the visible light spectrum. At 20 ˚C, the sample is liquid and transparent to the eyes based on the visual inspection as shown in the inset picture of Supplementary Fig 2. The transmittance of the sample was measured as a function of the wavelength (Supplementary Fig 2). The results show that this liquid sample is transparent to light over the whole visible light spectrum.

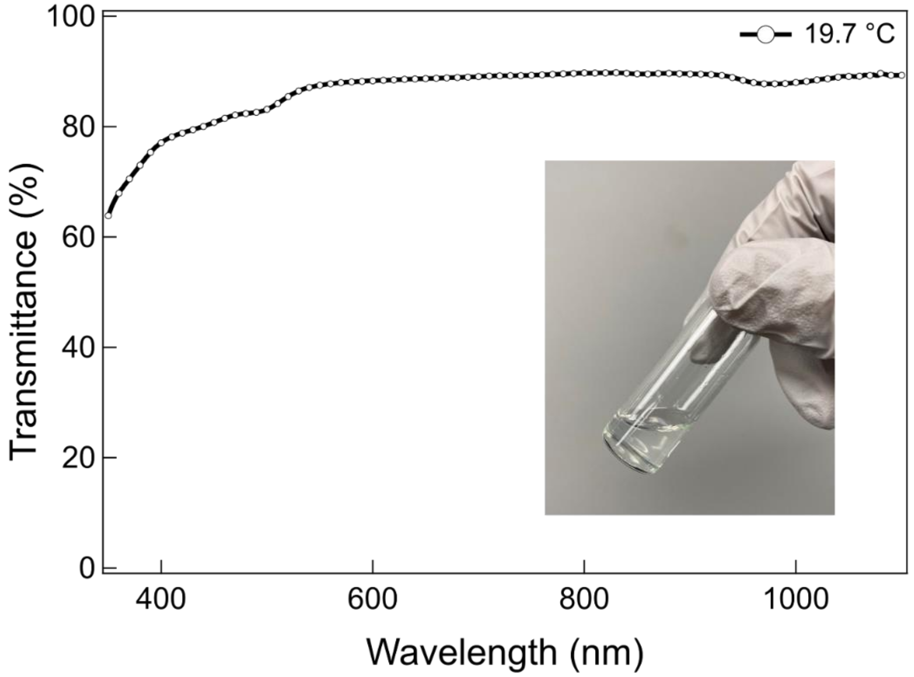

Supplementary Fig 2. Transmittance measurement of the sample in the liquid state at 20˚C. An identical sample is used as that shown in Figure 1 in the main text. The inset picture shows the appearance of the transparent sample in the liquid state.

Besides the dynamically tunable coloration for the whole visible light spectrum shown in Figure 1 of the main text, the transmitted light can be modulated by the SeedGel sample in both the ultraviolet and infrared region by changing temperatures (Supplementary Fig 3).

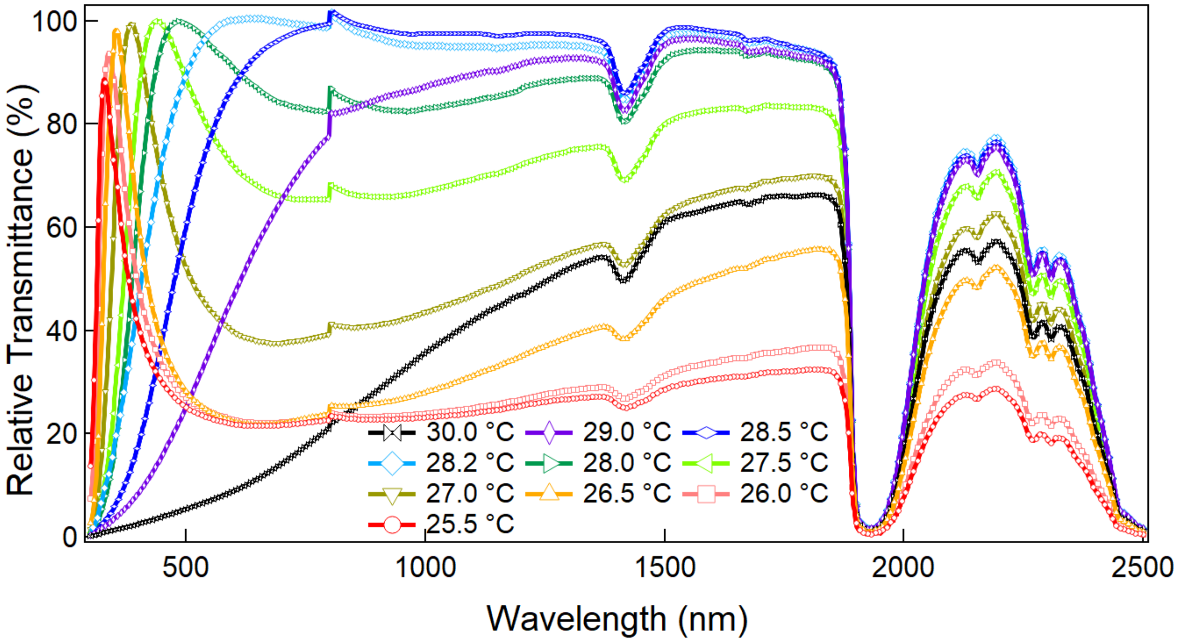

Supplementary Fig 3. The relative transmittance of SeedGel as a function of wavelength that covers UV-visible to the near-infrared range at different temperatures. The spectra are normalized to the spectrum of the liquid state sample measured at 20 ˚C. The relative transmittance is obtained by dividing the highest transmittance using the normalized spectra.

3. Modeling of Ultra-small angle neutron scattering (USANS) and small angle neutron scattering (SANS) data

Supplementary Fig 4 (a) shows the USANS scattering profiles of SeedGel measured at 27 ˚C, 28 ˚C, 29 ˚C, and 30 ˚C and their corresponding fittings using the Teubner-Strey model. The model has been widely used to describe bicontinuous structures and Sasview software was used to obtain the fitting results. [3–6] Teubner-Strey model (Eq-1 ~ Eq-4) is used to fit the scattering curve here. The parameters of periodicity (d), correlation length (ξ), and the contrast between the two domains ($(\Delta\rho)^2$) are allowed to vary, while other parameters are fixed as constants. φ_a is the volume fraction of one of the domains, which could be either the particle domain or the solvent domain. The volume fraction of the particle domain is determined from the ratio of the nominal particle concentration (about 24.3 %) over the local particle concentration in the particle domain. The local particle concentration in the particle domain is obtained from the fitting of SANS data at the high-q region, which is discussed in the next paragraph. Based on the fitting results listed in Supplementary Tab 1, the periodicity of SeedGel at 30 ˚C is about 3.4 µm. Consistent with the unchanged peak position in the temperature range between 27 ˚C and 30 ˚C, the periodicity of the bicontinuous domains of around 3.4 µm is observed within the temperature range that SeedGel exhibits dynamically tunable optical properties. The slight intensity change at varied temperatures is due to the solvent exchange between domains, which affects the contrast between the bicontinuous domains.

$$I(q) = \frac{8\pi\varphi_a(1-\varphi_a)(\Delta\rho)^2 c_2/\xi}{a_2+c_1 q^2+c_2 q^4} \qquad \text{Eq-1}$$

$$c_1 = -2\xi^2(\frac{2\pi\xi}{d})^2 + 2\xi^2 \qquad \text{Eq-2}$$

$$c_2 = \xi^4 \qquad \text{Eq-3}$$

$$a_2 = [1 + (\frac{2\pi\xi}{d})^2]^2 \qquad \text{Eq-4}$$

To obtain the local particle concentration in the particle domain, the SANS results are fitted using sphere model with the Hayter-Penfold method at different temperatures (Supplementary Fig 4(b)). [7,8] Static decoupling approximation (β-approximation) is used to account for the polydispersity of

the nanoparticles. [9] The radius of the nanoparticles is assumed to follow Gaussian distribution. Its polydispersity is defined as the standard deviation over the mean of the distribution, which is fixed at 0.1. The scattering length density of the solvent, volume fraction of particles in the particle domain, and the charge on the particle surface are left as variables of the fitting. All the rest of the parameters are fixed as constants. The radius of the sphere is determined by an independent experiment on a dilute sample with 0.5 % volume fraction silica nanoparticles dispersed in water. [1] The radius used in the fitting is 13.3 nm. The volume fractions of the particles in the particle domain at different temperatures are summarized in Supplementary Tab 1. It is clear that the local volume fractions of the particles in the particle domain stay around 40 % for all four temperatures, which is much higher than the particle concentration in the liquid dispersion state (about 24 %).

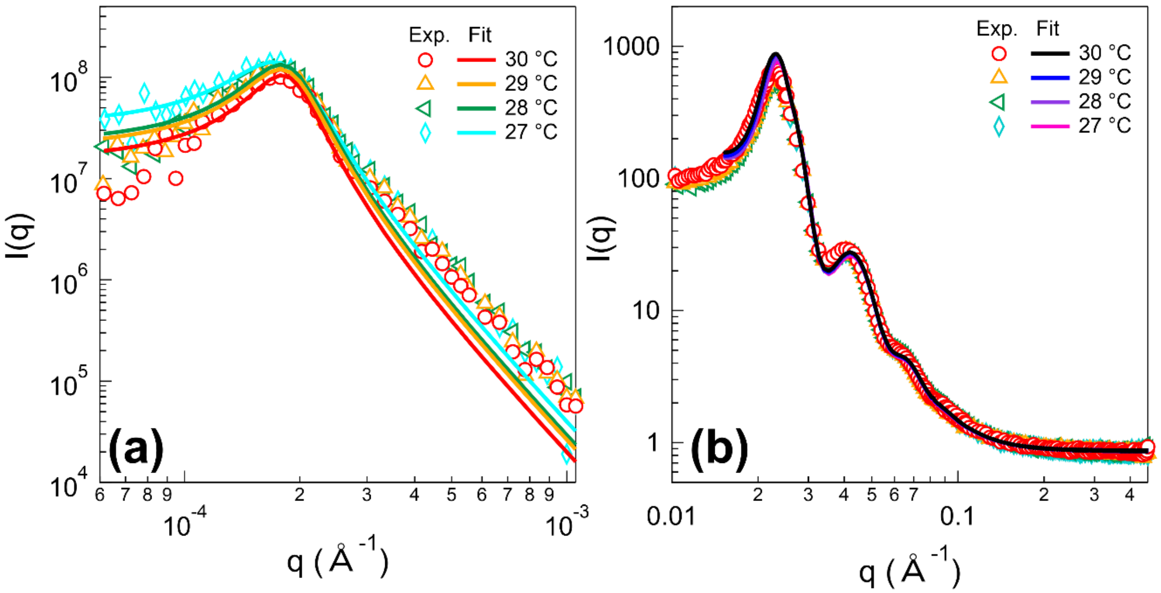

Supplementary Fig 4. Fittings to USANS and SANS results. (a) Desmeared USANS scattering profiles of SeedGel collected at 27 ˚C, 28 ˚C, 29 ˚C, and 30 ˚C. The Teubner-Strey model is used to fit the experimental data. (b) SANS scattering profiles of SeedGel collected at 27 ˚C, 28 ˚C, 29 ˚C, and 30 ˚C. Sphere model with Hayter-Penfold method is used to fit the SANS results between 0.15 Å^{-1} and 0.461 Å^{-1}. The error bars represent one standard deviation and are often smaller than the symbol size.

Supplementary Tab 1. Periodicity obtained from the Teubner-Strey model fitting of the desmeared USANS results at different temperatures. Local particle volume concentration in the particle domain estimated from the high-q SANS data fitting using sphere model with Hayter-Penfold method. The volume fractions of the particle domain and the solvent domain are determined based on the local particle concentration in the particle domain.

Temperature (˚C)	Periodicity (μm)	Local Particle Concentration in Particle Domain (%)	Volume Fraction of Particle Domain (%)	Volume Fraction of Solvent Domain (%)
30	3.4	39.0	62.8	37.2
29	3.4	39.2	62.6	37.4
28	3.4	40.4	60.6	39.4
27	3.6	40.9	59.8	40.2

4. **Small angle X-ray scattering (SAXS) measurements rule out the formation of colloidal crystals.**

Photonic crystals are known to scatter light at a certain wavelength that is related to their lattice parameter. Typically, they have ordered structures with an inter-particle distance of a few hundred nanometers (comparable to the wavelength of light) so that the materials can preferentially reflect light at a certain wavelength.

Ultra-small angle X-ray scattering (USAXS) and small angle X-ray scattering (SAXS) are used to investigate the structures of particles in the SeedGel sample at different temperatures (Supplementary Fig 5). Due to the large contrast between the silica particles and solvent, the SAXS scattering pattern, I(q), is mainly sensitive to particle structures. Here, q is the scattering wave vector with $q = (4\pi/\lambda)\sin(\theta/2)$, where λ is the wavelength of X-ray and θ is the scattering angle. The inter-particle distance estimated from the inter-particle structure factor peak is about 30 nm close to the diameter of the silica nanoparticles. There are no sharp peaks that are typically observed for crystalline samples. Thus, the silica nanoparticles are packed in a disordered state without any long-range periodic structure. The results are consistent with the SANS data in Figure 2 (a) in the main text.

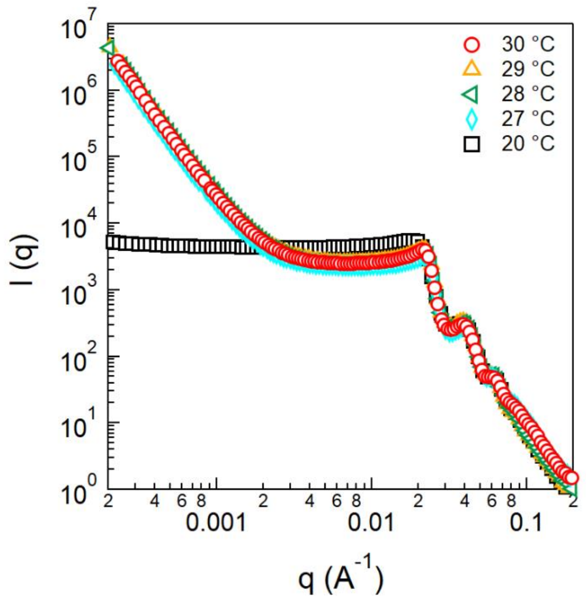

Supplementary Fig 5. SAXS profiles of SeedGel within the temperature range with modulated visible light transmission.

5. Estimation of the averaged refractive index as the function of the wavelength for both the particle and solvent domains.

To calculate the averaged refractive index of both the particle and solve domains, the composition of each domain needs to be estimated first.

Our SAXS experiments show that the inter-particle peak intensity at ~ 0.02 Å^{-1} remains the same in the gel state. Thus, the particle packing is not affected by the solvent composition change in the particle domain as the SAXS is sensitive to the particle structures, but it is less sensitive to the solvent composition change.

To obtain the solvent composition in each domain, we perform a SANS experiment as it can be sensitive to the solvent composition. To achieve this, we adjust the scattering length density (SLD) of water to be the same as that of silica with the volume ratio between H_2O and D_2O to be 42: 58. When there is a 2,6-lutidine exchange between particle domain and solvent domain, the scattering contrast (SLD difference) between silica particle and the surrounding solvent is altered. By analyzing the contrast change, the solvent composition in the particle domain can be estimated. Based on the mass balance, we can further estimate the composition in the solvent domain.

The SANS results are presented in Supplementary Fig 6. In the gel state, the scattering intensity at $q > 0.02$ Å^{-1} is mainly due to the difference of SLD between silica and the solvent around the particles in the particle domain. The SLD of the silica particles remains the same at all probed temperatures. However, the SLD of the solvent surrounding the particles is affected by the solvent composition. In the liquid state, the SLD of the solvent is a mixture of lutidine and water. In the

gel state, the lutidine concentration is reduced in the solvent of the particle domain. The SLD difference between silica particles and the surrounding solvent becomes much smaller as the SLD of water is matched to that of silica. Therefore, the scattering intensity of the inter-particle peak at ~0.02 $Å^{-1}$ is greatly reduced in the gel state compared to that in the liquid state (20 ˚C).[1]

Ramping up the temperature further decreases the lutidine concentration and increases the water concentration in the particle domain. Using a method discussed previously,[1] it is calculated that the mass fraction of lutidine could decrease from 15.3 % to 9.7 % in the particle domain when the temperature is increased from 27.5 ˚C to 30 ˚C. The lutidine concentration in the solvent domain can be estimated based on the mass balance. The amounts of different components in both domains are listed in Supplementary Tab 2. It is important to note that the fraction of each component is normalized to the total mass of solvent in each domain. In the calculation, the concentration of silica nanoparticles in the particle domain is fixed at a volume fraction of 39.1 % based on the SAXS results.

Supplementary Tab 2. The calculated mass fraction of lutidine and water in both particle and water domain.

	Particle Domain		Solvent Domain	
	Lutidine (%)	Water (%)	Lutidine (%)	Water (%)
27.5 ˚C	15.3	84.7	41.6	58.4
30.0 ˚C	9.7	90.3	47.3	52.7

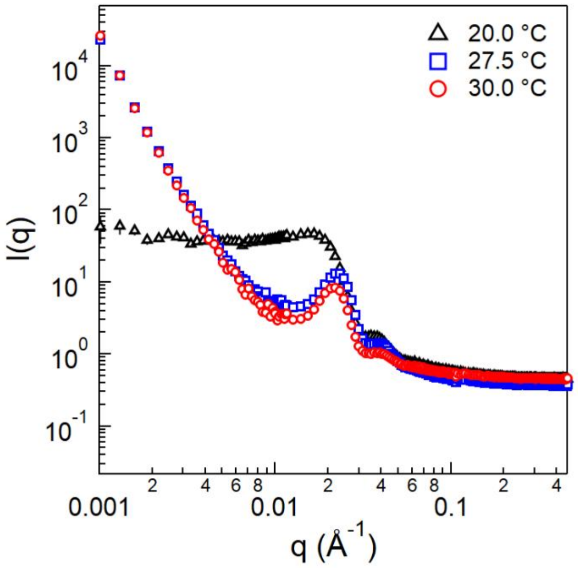

Supplementary Fig 6. SANS results of SeedGel with the SLD of the water-phase matched that of silica at different temperatures. The error bars represent one standard deviation and are often smaller than the symbol size.

With the estimated solvent composition of each component in both domains, the refractive index can be estimated using the Lorentz-Lorenz formula (Eq-5). The mass density (ρ), Avogadro's number (N), molecular polarity (ϑ), and molecular weight (M) are used to obtain the refractive index (n). In this equation, F is a corrective term related to the mixing of lutidine and water close to its critical temperature. The refractive index of the binary solvent within this temperature range has been investigated and documented in the literature.[10] The mixture of water and lutidine shows a strong wavelength (λ) and temperature (T) dependence, which is described in Eq-6.[10] In Eq-6, c_l is the mass fraction of lutidine, whereas T_0 is 33.5 ˚C and λ_0 is 632.8 nm. The term $B\{c_l\}$ can be calculated with Eq-7. The reported value of the derivative of refractive index with respect to temperature ($\frac{dn}{dT}$) are tabulated in the reference.[10] With the reported values of the parameters in the literature, the refractive index of the water-lutidine mixture can be calculated as a function of wavelength at a given temperature at the lutidine mass fractions of 0.1033, 0.2087, 0.4061, and 0.5064.[10] A linear extrapolation is used to estimate the refractive index of the binary solvents at the actual concentrations corresponding to that in both particle and solvent domains estimated from the SANS results (Supplementary Tab 2).

The refractive index of the particle domain can be obtained with a knowledge of the refractive index of the binary solvent and silica particles using the Lorentz-Lorenz formula (Eq-8). It is reasonable to assume that the refractive index of silica is independent of temperature change between 26 ˚C and 30 ˚C. The refractive indices of both the particle domain and solvent domain depend on temperature because the solvent exchanges between domains result in a change of solvent composition. In the calculation, the binary solvent is treated as one component and silica nanoparticles are treated as a second component. Their molecular polarities are calculated from their corresponding refractive indices using Eq-8. The mixing rule described in Eq-9 is used to determine the averaged molecular polarity when silica particles are mixed with the binary solvent, where c is the mole fraction of one of the components.

It is worth mentioning that the absolute value of the refractive index of silica strongly depends on its polymorph.[11,12] Fused silica and quartz have different values of refractive index, which are plotted as a function of wavelength in Supplementary Fig 7(a). (The difference is about 0.086 over the studied wavelength range.) However, it is important to point out that the two polymorphs exhibit similar wavelength dependence. By vertically shifting the refractive index of fused silica by 0.086, the curve of fused silica almost overlaps with that of quartz.

The refractive index of the particle domain in our SeedGel is calculated using both polymorphs of silica. The results are shown in Supplementary Fig 7(b). There is no observable discrepancy after shifting the refractive index of one curve by 0.034 as shown in Supplementary Fig 7(b). The polymorph of the silica does not affect the wavelength dependence of the refractive index of the particle domain. Therefore, the type of silica we use to estimate the refractive index does not affect the curvature of the curve. Hence, we used the refractive index of fused silica for the rest of the calculations.[12]

The dynamical tunability of optical transmission of the SeedGel is mainly driven by the different wavelength dependence of the refractive index between the particle domain and solvent domain. The volume fraction of silica particles in the particle domain is 39.1 %. For the binary solvent in the particle domain, the mass concentration of lutidine and water is 15.3 %, 84.7 % respectively. These particle and solvent concentrations correspond to the compositions of the particle domain at 27.5 ˚C.

As the values of the refractive index of the binary solvent have been well studied in literature[10], the final value of the refractive index of the particle domain is calibrated by vertically shifting the calculated value to match that of the solvent domain at the wavelength of the transmittance peak. This is because when the refractive indices of the two domains are the same, the transmittance should reach the maximum value as the scattering intensity is minimized due to the lack of the scattering contrast. The refractive index of the particle domains in Supplementary Fig 8(a) is shifted vertically by 0.0229. This is based on the experimental fact that the sample is transparent for light with short wavelengths at low temperatures. The obtained refractive indices of the particle domain and the solvent domain as a function of wavelength are shown in Supplementary Fig 8(a) and Supplementary Fig 8(b), respectively.

Importantly, the wavelength dependence (curvature) of the two domains is different. When increasing temperature, the refractive index of the particle domain decreases, and that of the solvent domain increases. Due to the different wavelength dependence of the refractive index, the matching wavelength, where the refractive indices of both domains are identical, shifts from a shorter wavelength to a long wavelength. This is further discussed in detail in Section 7 of the supporting information. By changing temperature from 30 ˚C to 27.5 ˚C, the combined change in the refractive index of these two domains is approximately 0.0135.

$$\frac{(n^2-1)}{(n^2+2)} = \left(\frac{4\pi N}{3}\right)\left(\frac{\theta\rho}{M}\right)(1+F) \qquad \text{Eq-5}$$

$$n\{c_l, T, \lambda\} = n\{c_l, T_0, \lambda_0\} + B\{c_l\}(\lambda^{-2} - \lambda_0^{-2}) + \left(\frac{dn}{dT}\right)\{c_l, T_0, \lambda_0\}(T - T_0) \qquad \text{Eq-6}$$

$$B\{c_l\} = 10^{-15}(3.3 + 3.3c_l + 1.8c_l^2) \qquad \text{Eq-7}$$

$$\frac{(n^2-1)}{(n^2+2)} = \left(\frac{4\pi N}{3}\right)\left(\frac{\theta\rho}{M}\right) \qquad \text{Eq-8}$$

$$\frac{\theta\rho}{M} = \frac{c\theta_1\rho_1}{M_1} + \frac{(1-c)\theta_2\rho_2}{M_2} \qquad \text{Eq-9}$$

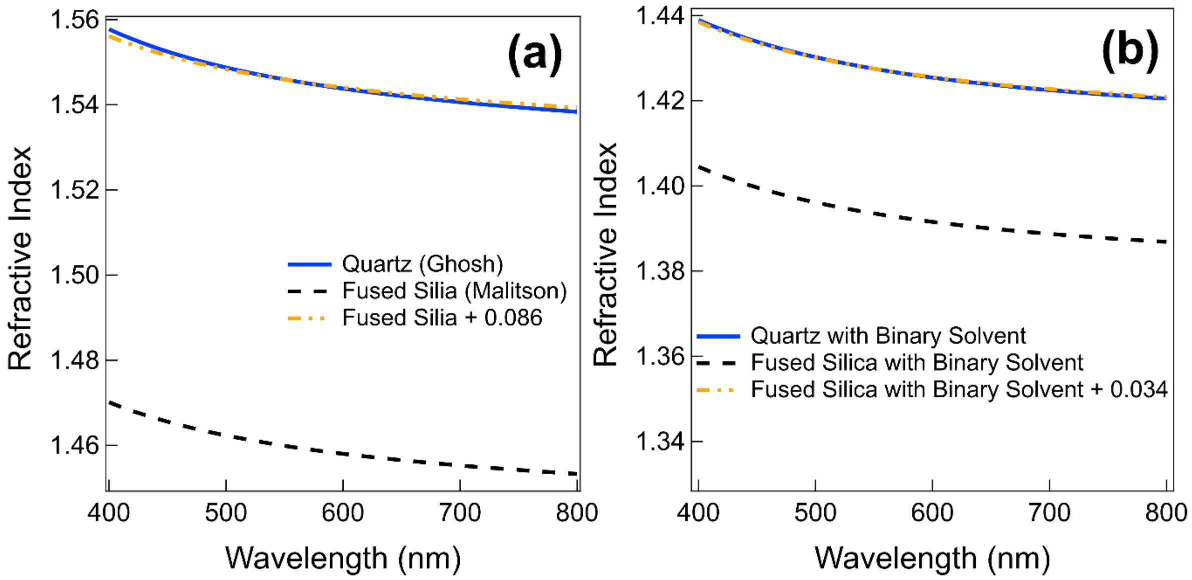

Supplementary Fig 7. The wavelength-dependent refractive indexes. (a) Refractive index of quartz and fused silica as a function of wavelength.[11,12] They show similar wavelength dependence. By shifting the curve of fused silica by 0.086, the refractive index of quartz and silica almost overlap with each other. (b) The refractive index of the particle domain (silica volume fraction of 39.1 %) is estimated as a function of wavelength using both quartz and fused silica. The binary solvent is composed of 15.3 % of lutidine in water by mass. By shifting the mixture using fused silica by 0.034, two curves overlap with each other.

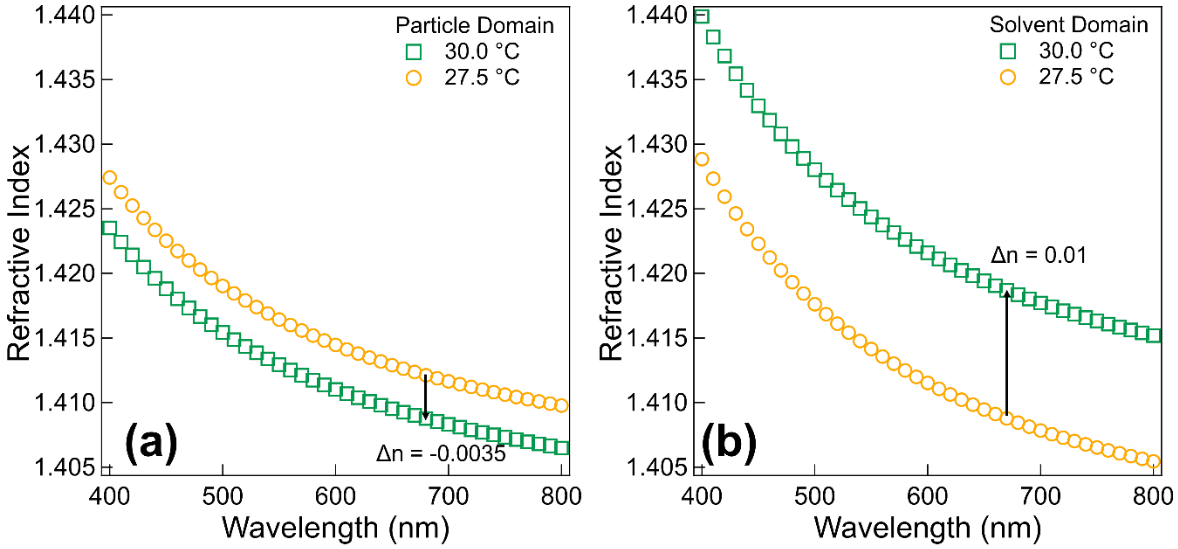

Supplementary Fig 8. The wavelength-dependent refractive index of both particle domain and solvent domain. (a) Estimated refractive index of the particle domain as a function of wavelength at 30 ˚C and 27.5 ˚C based on the Lorentz-Lorenz formula.[10] (b) Calculated refractive index of the

solvent domain as a function of wavelength at 30 ˚C and 27.5 ˚C. The y-axis is fixed with the same range for (a) and (b) to show the different wavelength dependence of the refractive index between the two domains.

6. **Static light scattering measurement also confirms the wavelength-dependent refractive index matching between the particle and the solvent domains.**

The static light scattering (SLS) measurements were performed with a modified Brookhaven BI-200SM using a wavelength of 532 nm laser light from a Coherent VERDI diode-pumped solid-state laser operating in TEM00 mode. Glan-laser polarizer and analyzer (Thorlabs) were used under the vertical polarizer and vertical analyzer conditions. The laser power was finely adjusted by neutral density filters, and a 1-mm pinhole was used before the photomultiplier. The scattered intensity $I(q)$ was collected between the scattering angles of 20° to 140° in 5° increments enabled by the precision goniometer and detector arm, corrected for reflection, angle-dependent scattering volume variation, and refraction by standard methods, and subsequently plotted as a function of scattering wave vector q, where $q = (4\pi n/\lambda)\ \sin(\theta/2)$. Here, n is the refractive index. All samples were thermostatically controlled by a recirculating bath to control the temperature of the decalin index matching bath. The sample temperature was monitored by a platinum resistance thermocouple placed within the vat, with a precision of ± 0.1 °C.

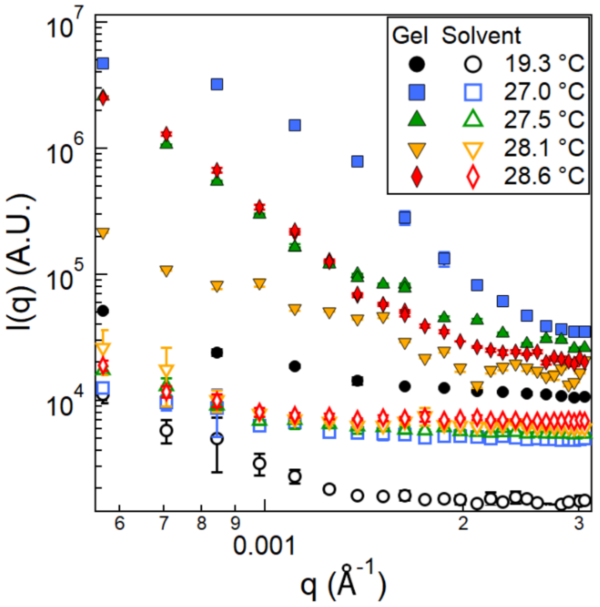

Supplementary Fig 9. Static light scattering results of SeedGel performed at different temperatures with a 532 nm laser. The error bars in the figure represent one standard deviation and are often smaller than the symbol size.

The SLS results of a SeedGel sample are shown in Supplementary Fig 9. Note that the wavelength of the laser is 532 nm. The flat scattering curves of the solvent mixture (lutidine and water) at different temperatures indicate that the solvent mixture itself does not form structures at the length

scale corresponding to the q-range probed by the SLS. In contrast, the SeedGel sample exhibits a large scattering intensity at 27 ˚C. Further increasing the temperature reduces the scattering intensity. The scattering pattern shows the lowest intensity at 28.1 ˚C. At 28.6 ˚C, the scattering intensity increases again. This is consistent with the proposed mechanism in the previous section. The strong scattering at 27 ˚C is due to the mismatch of the refractive index of the two domains at this particular wavelength. Further increasing the temperature shifts the matching wavelength closer to the used laser wavelength and reduces the contrast between the two domains. The lowest static light scattering intensity occurs at 28.1 ˚C where the matching point of the wavelength of these two domains is very close to the laser wavelength. Further increasing the temperature shifts the matching wavelength and increases the contrast at the wavelength of the laser resulting in increased scattering intensity at 28.6 ˚C.

This trend measured by the light scattering experiment is consistent with the transmission results in Figure 1 in the main text. By increasing the temperature from 27 ˚C, the transmission of 532 nm light gradually increases, indicating a reduced contrast between domains. It results in a decrease of the static scattering intensity from 27 ˚C to 28.1 ˚C. The transmittance of 532 nm light reaches a maximum close to 28.1 ˚C, suggesting a matched refractive index between the domains.

7. Calculation of light transmittance of a SeedGel sample

It is well known that the transmitted intensity of light (*I*) can be related to the incoming intensity (I_0) by $\frac{I}{I_0} = e^{-\Sigma d}$, where Σ is the macroscopic scattering cross section and *d* is the sample path length. For a general case, Σ depends on both the scattering and absorption cross section. Since the temperature effect of the light absorption is negligible within the studied temperature range, the temperature-dependent transmittance in our SeedGel samples is thus solely due to the change of the scattering cross section. And the macroscopic scattering cross section is proportional to the contrast, $(\Delta\rho)^2$, where Δρ is the refractive index difference between two domains.

Thus the light transmittance can be described by Eq-10. Here, Δρ can be estimated using Eq-11, which is closely related to the refractive indices of the particle domain (n_p) and solvent domain (n_s). [13] Here, λ in Eq-11 is the wavelength of light. '*A*' is related to the structure of the SeedGel during the studied temperature range. As the bicontinuous domain structures remain the same during the experiment, '*A*' can be considered as a constant value with the investigated temperature range. Here, a fixed value of 6.54×10^{10} Å^3 is chosen for '*A*' as it results in a very good agreement of the estimated transmittance with the experimental results during the investigated temperature range. Note that for all calculated transmittance at different temperatures, '*A*' is a fixed constant.

$$\frac{I}{I_0} = e^{-\Sigma d} \propto e^{-(\Delta\rho)^2 A d} \qquad \text{Eq-10}$$

$$\Delta\rho = \frac{3\pi n_s^2}{\lambda^2} \frac{(\frac{n_p}{n_s})^2 - 1}{(\frac{n_p}{n_s})^2 + 2} \qquad \text{Eq-11}$$

Increasing the temperature decreases n_p and increases n_s. Because the wavelength dependence of the two domains is different from each other, the refractive index between the two domains only matches at a single wavelength at a given temperature. Heating the SeedGel sample results in a shift of the matching point towards the longer wavelength. As a result, the position of the transmittance peak shifts to a longer wavelength when increasing the temperature.

The theoretically calculated transmission spectra together with the estimated refractive index of the two domains are shown in the same figure (Supplementary Fig 10). The results in panels (a) to (h) correspond to the sample from the low temperature (Supplementary Fig 10 (a)) to the high temperature (Supplementary Fig 10(h)). The refractive index of the solvent domain plotted in Supplementary Fig 10 (a) is based on the estimated results of SeedGel at 27.5 ˚C. Since the effect of the temperature change is only to slightly shift the whole wavelength-dependent refractive index curve in the vertical direction, only the refractive index of the particle domain is adjusted from Supplementary Fig 10 (a) to Supplementary Fig 10 (h) to simplify the calculation. This is reasonable as only the refractive index difference between the two domains matters when estimating the light transmission.

The peak transmittance (100 %) occurs at the wavelength that the refractive index of the two domains match. Light at other wavelengths is scattered away by the sample since the large domain size of the SeedGel scatters strongly even with a very minor difference of the refractive index. Based on the estimation, the relative difference of the refractive index between the two domains only needs to change by 0.007 to shift the transmission peak across the whole visible spectrum. Based on the actual composition of each component estimated from SANS measurements in both domains (Supplementary Fig 6), the total change of refractive index difference can be as large as 0.0135 when the temperature changes from 27.5 ˚C to 30 ˚C (Supplementary Fig 8 in the Supporting Information). This large change of the refractive index also allows the SeedGel to finely control the peak position of the light transmittance in the ultraviolet and infrared region.

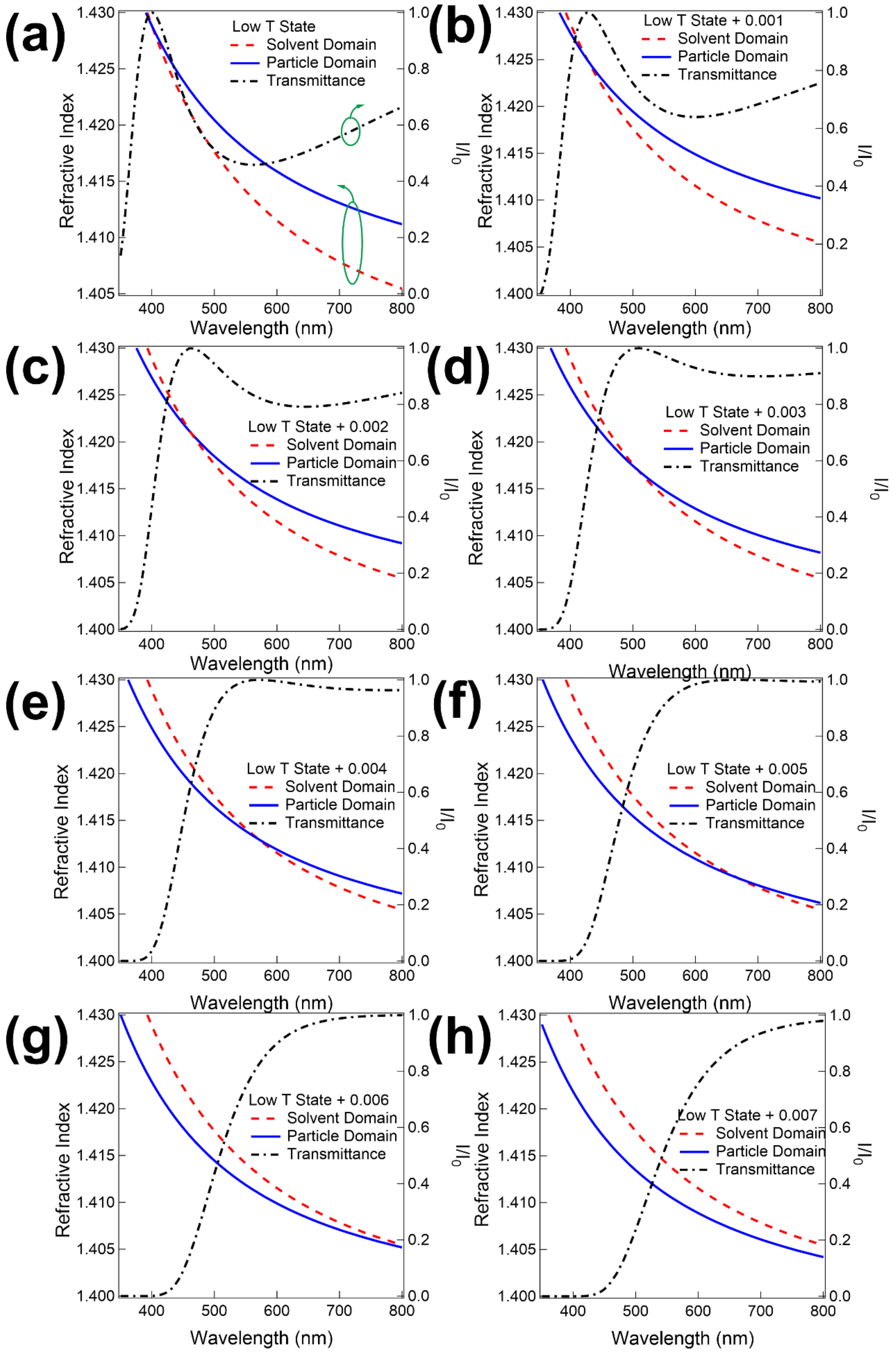
(a)
Low T State
Solvent Domain
Particle Domain
Transmittance
Refractive Index
I/I0
Wavelength (nm)
(b)
Low T State + 0.001
Solvent Domain
Particle Domain
Transmittance
Refractive Index
I/I0
Wavelength (nm)
(c)
Low T State + 0.002
Solvent Domain
Particle Domain
Transmittance
Refractive Index
I/I0
Wavelength (nm)
(d)
Low T State + 0.003
Solvent Domain
Particle Domain
Transmittance
Refractive Index
I/I0
Wavelength (nm)
(e)
Low T State + 0.004
Solvent Domain
Particle Domain
Transmittance
Refractive Index
I/I0
Wavelength (nm)
(f)
Low T State + 0.005
Solvent Domain
Particle Domain
Transmittance
Refractive Index
I/I0
Wavelength (nm)
(g)
Low T State + 0.006
Solvent Domain
Particle Domain
Transmittance
Refractive Index
I/I0
Wavelength (nm)
(h)
Low T State + 0.007
Solvent Domain
Particle Domain
Transmittance
Refractive Index
I/I0
Wavelength (nm)

Supplementary Fig 10. Transmittance calculated from the refractive indexes of both the particle and solvent domains as a function of wavelength. The combined change of the refractive index between the two domains ranges from 0 to 0.007 in (a) ~ (h), respectively.

In summary, four characteristics of this SeedGel render its exciting optical properties. These characteristics are: 1) the different wavelength dependence of the refractive index between the particle and solvent domains; 2) the formation of large-scale (micrometer) structures that scatter light strongly when the refractive index is not matched well between two domains; 3) the refractive index can be effectively manipulated using the binary solvent to achieve dynamic tunability; 4) each domain has a uniform structure. For 1), the difference of the wavelength dependence of the refractive index is introduced by the particles, because silica particles have a different wavelength dependence of the refractive index than that of the binary solvent in this material. For 2), SeedGel forms micrometer-sized domains that can scatter light strongly even with a slight difference of the refractive index between these two domains. For 3), the miscibility of the binary solvent can be used to control the refractive index of both domains in response to a temperature change. For 4), particles in the particle domain with a nominal diameter of ≈ 30 nm are highly charged, so the particles are uniformly dispersed in the particle domain without forming heterogeneous structures with dimensions comparable to the light wavelength.

8. Controlling the FWHM of the light transmittance peak.

The wavelength selectivity is due to scattering and is described by Eq-10: $\frac{I}{I_0} \propto e^{-(\Delta\rho)^2 Ad}$. Thus, the path length can be used to control the full width at half maximum (FWHM) of the transmitted light and enhance the color clarity. By increasing the sample path length from 1mm to 10mm, the measured FWHM can be significantly decreased to 44 nm (experimental results shown in Supplementary Fig 11). Further, we performed a theoretical calculation using the parameters in Supplementary Fig 10 except for the sample thickness. Supplementary Fig 12 shows the calculated results of the two different sample thicknesses. The transmitted light could improve the FWHM from about 100 nm to about 30 nm when the path length is increased by 10 times. This theoretical calculation is consistent with the experimental results shown in Supplementary Fig 11.

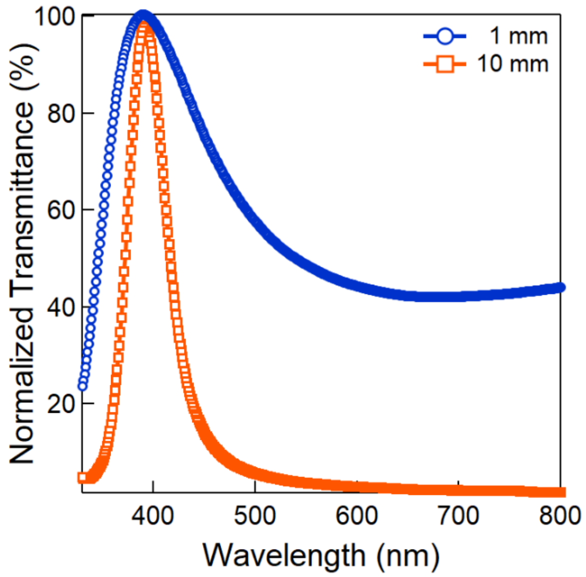

Supplementary Fig 11. Measured transmittance of SeedGel with 1 mm and 10 mm pathlengths as a function of wavelength. The transmittance spectra are normalized to that of the same sample measured at 20 ˚C. The increase in path length sharply reduces the FWHM to 44 nm.

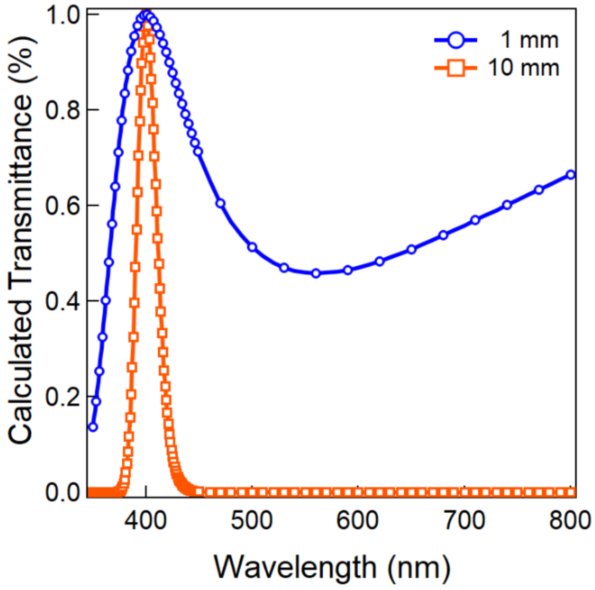

Supplementary Fig 12. Calculated transmittance of SeedGel with different path lengths.

9. **Customizable color transition temperature.**

It is shown that the gel transition temperature of the SeedGel can be controlled by simply switching to different binary solvents. [1] Thus, the functional temperature of the color effect can be controlled

by using different binary solvents too. This offers tremendous flexibility for the applications of these materials at different temperatures.

Supplementary Fig 13 shows four examples of adjusting the gelation temperature using four different binary solvent pairs. The measurements of the light transmission as a function of the wavelength confirm that they all show similar optical properties as demonstrated in Figure 1(e) of the main text. A broad range of color transition temperatures is covered from 15 ˚C to about 100 ˚C. By using a binary solvent pair of water/2,4-lutidine (an isomer of 2,6-lutidine), the transition temperature can be shifted to below 20 ˚C. The solvent pair of water/3-methylpyridine increases the temperature transition range to close to 40 ˚C. SeedGel prepared with the deuterated version of the solvent (water/ deuterated 3-methylpyridine) shifts the color transition temperature to about 60 ˚C. Using the isomers further increases the color transition temperature to around 80 ˚C. It is important to note that the four examples here are not intended to exhaust the wide variety of solvent pairs that could be used in SeedGel. Rather, the color transition temperatures are designed to space apart to show the wide range of customizability that is promising for various applications.

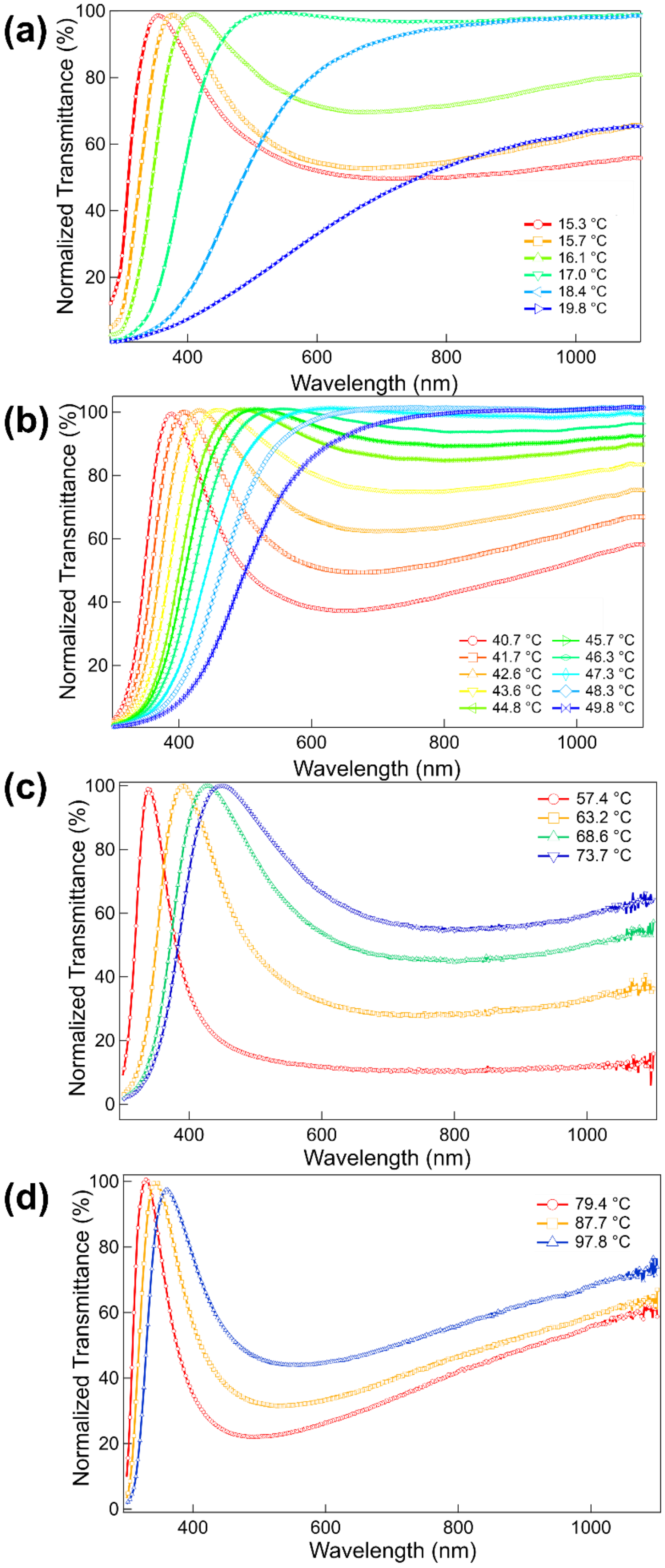
(a)
Normalized Transmittance (%)
Wavelength (nm)
15.3 °C
15.7 °C
16.1 °C
17.0 °C
18.4 °C
19.8 °C
(b)
Normalized Transmittance (%)
Wavelength (nm)
40.7 °C
41.7 °C
42.6 °C
43.6 °C
44.8 °C
45.7 °C
46.3 °C
47.3 °C
48.3 °C
49.8 °C
(c)
Normalized Transmittance (%)
Wavelength (nm)
57.4 °C
63.2 °C
68.6 °C
73.7 °C
(d)
Normalized Transmittance (%)
Wavelength (nm)
79.4 °C
87.7 °C
97.8 °C

Supplementary Fig 13. Transmittance measurements of SeedGel samples prepared with different binary solvents. The binary solvent include (a) water/2,4-lutidine, (b) water/ hydrogenated 3-methylpyridine, (c) water/ deuterated 3-methylpyridine, (d) water/ 2-methylpyridine at different temperatures. The pathlength of the measurements is 1 mm. The volume percentages of water in the binary solvent are 70 %, 63.6 %, 63.6 %, 63.6 % in (a), (b), (c), and (d), respectively. The particle volume fractions in the total volume of the samples are (a) 24.5 % (b) 22.7 % (c) 22.7 % and (d) 22.7 %, respectively.

Movie S1.

Optical transmission through colloidal gel with bicontinuous structures during cooling.

Acknowledgments:

This work benefited from the use of the SasView application, originally developed under NSF Award DMR-0520547. SasView also contains code developed with funding from the EU Horizon 2020 program under the SINE2020 project Grant No 654000. Certain commercial equipment, instruments, or materials (or suppliers, or software, ...) are identified in this paper to foster understanding. Such identification does not imply recommendation or endorsement by the National Institute of Standards and Technology, nor does it imply that the materials or equipment identified are necessarily the best available for the purpose.

References:

1. Xi, Y., Lankone, R. S., Sung, L.-P. & Liu, Y. Tunable thermo-reversible bicontinuous nanoparticle gel driven by the binary solvent segregation. *Nat. Commun.* **12**, 910 (2021).
2. Xi, Y. *et al.* Controlling Bicontinuous Structures through a Solvent Segregation-Driven Gel. *Langmuir* **37**, 2170–2178 (2021).
3. Teubner, M. & Strey, R. Origin of the scattering peak in microemulsions. *J. Chem. Phys.* **87**, 3195–3200 (1987).
4. Schubert, K. -V., Strey, R., Kline, S. R. & Kaler, E. W. Small angle neutron scattering near Lifshitz lines: Transition from weakly structured mixtures to microemulsions. *J. Chem. Phys.* **101**, 5343–5355 (1994).
5. Endo, H. *et al.* Effect of amphiphilic block copolymers on the structure and phase behavior of oil–water-surfactant mixtures. *J. Chem. Phys.* **115**, 580–600 (2001).
6. M. Doucet; et al. SasView Version 5.0.4, Zenodo, DOI:10.5281/zenodo.4467703.
7. Hayter, J. B. & Penfold, J. An analytic structure factor for macroion solutions. *Mol. Phys.* **42**, 109–118 (1981).
8. Hansen, J.-P. & Hayter, J. B. A rescaled MSA structure factor for dilute charged colloidal dispersions. *Mol. Phys.* **46**, 651–656 (1982).

9. Kotlarchyk, M. & Chen, S. Analysis of small angle neutron scattering spectra from polydisperse interacting colloids. *J. Chem. Phys.* **79**, 2461–2469 (1983).

10. Jayalakshmi, Y., Van Duijneveldt, J. S. & Beysens, D. Behavior of density and refractive index in mixtures of 2,6-lutidine and water. *J. Chem. Phys.* **100**, 604–609 (1994).

11. Ghosh, G. Dispersion-equation coefficients for the refractive index and birefringence of calcite and quartz crystals. *Opt. Commun.* **163**, 95–102 (1999).

12. Malitson, I. H. Interspecimen Comparison of the Refractive Index of Fused Silica*,†. *J. Opt. Soc. Am.* **55**, 1205 (1965).

13. Xu, R. *Particle Characterization: Light Scattering Methods*. (Springer, 2002).
