# Supplementary material for: Finely tunable dynamical coloration using bicontinuous micrometer-domains
Source: Nat Commun. 2022 Jun 24;13:3619. doi: 10.1038/s41467-022-31020-0 (PMC9232638; doi:10.1038/s41467-022-31020-0)
Supplement: Supplementary file 2 — Description of Additional Supplementary Files [file 41467_2022_31020_MOESM2_ESM.pdf]

File Name: Supplementary Movie 1

Description: Optical transmission through SeedGel, colloidal gel with bicontinuous structures, during cooling.
